# Sedimentary biomarkers and bone specimens reveal a history of prehistoric occupation on Somerset Island (Arctic Canada)

**DOI:** 10.1098/rspb.2023.2915

**Published:** 2024-07-10

**Authors:** Lauren R. Gallant, Kathryn E. Hargan, Linda E. Kimpe, Neal Michelutti, Christopher Grooms, James M. Savelle, John P. Smol, Jules M. Blais

**Affiliations:** ^1^ Department of Biology, University of Ottawa, Ottawa, ON K1N 6N5, Canada; ^2^ Department of Biology, Memorial University of Newfoundland, St. John’s, NL A1B 3X9, Canada; ^3^ Department of Biology, Paleoecological Environmental Assessment and Research Lab (PEARL), Queen’s University, Kingston, ON K7L 3N6, Canada; ^4^ Department of Anthropology, McGill University, Montreal, Quebec H3A 2T7, Canada

**Keywords:** Thule, Dorset, sterols and stanols, metals, stable isotopes

## Abstract

Archaeological studies of pre-historic Arctic cultures are often limited to artefacts and architecture; such records may be incomplete and often do not provide a continuous record of past occupation. Here, we used lake sediment archives to supplement archaeological evidence to explore the history of Thule and Dorset populations on Somerset Island, Nunavut (Canada). We examined biomarkers in dated sediment cores from two ponds adjacent to abandoned Thule settlements (PaJs-3 and PaJs-13) and compared these to sediment cores from two ponds without past human occupation. Coprostanol and epicoprostanol, δ^15^N measurements, sedimentary chlorophyll *a* and the ratio of diatom valves to chrysophyte cysts were elevated in the dated sediment profiles at both sites during Thule and Dorset occupations. Periods of pronounced human impact during the Thule occupation of the site were corroborated by ^14^C-dated caribou bones found at both sites that identified intense caribou hunting between *ca* 1185 and 1510 CE. Notably, these sediment core data show evidence of the Dorset occupation from *ca* 200 to 500 CE at sites where archaeological evidence was heretofore lacking. We highlight the utility of lake sediments in assisting archaeological studies to better establish the timings, peak occupations and even lifestyle practices of the Dorset and Thule Arctic peoples.

## 1. Introduction

Canada’s High Arctic was home to several cultures including Pre-Dorset (*ca* 2500–500 BCE), Dorset (*ca* 500 BCE to *ca* 1250 CE) and Thule (*ca* 1200–1600 CE) people [1–4]. The Thule people, who are the direct ancestors of the present-day Inuit, were a group of whalers who arrived by the Bering Strait approximately 800 years ago [1]. The Thule specialized in hunting bowhead whales; one report estimated that they hunted approximately 18 523 whales from 1200 to 1500 CE in Greenland and the Canadian Arctic [2]. The whale bones were used to build both over-wintering structures, such as permanent and semi-permanent houses, as well as caches and kayak and umiak rests [1–3]. The Thule culture abandoned much of the High Arctic approximately 500 years ago; this abandonment is believed to be related to the decreased availability of bowhead whales owing to increased summer sea-ice cover during the Little Ice Age (*ca* 1400/1500–1850 CE) [2]. The Thule camps and settlements are still visible today; whale bones and house foundations are often observed near coastal freshwater ponds, where the Thule would have obtained both their drinking water (or freshwater ice) and mosses that were used to insulate their houses.

Before the Thule, the High Arctic was occupied by Pre-Dorset and Dorset peoples. Dorset occupation intensity declined between *ca* 750 and 1250 CE, and then they disappeared throughout the Arctic shortly thereafter [4]. No evidence exists to suggest that the Thule and Dorset were genetically related; however, the Thule did subsequently inhabit some of the same locations as the Dorset [4]. The Dorset people over-wintered in shallow dug-out dwellings [4,5]. Unlike the Thule, the Dorset did not have the technology (e.g. large boats, throwing harpoons or skin floats) to hunt whales [6]. Instead, the Dorset subsistence economy focused on smaller sea mammals, such as seals and walrus, and terrestrial mammals, such as caribou and musk-ox [6].

To date, much of the research on the first inhabitants in the High Arctic has been derived from archaeological studies using traditional approaches (e.g. settlement pattern analysis, artefact typologies and radiocarbon dating). For example, the number and type of Thule houses, weapons and other tools have been extensively studied at several sites [2,7–11]. However, archaeological investigations using traditional approaches may have limitations owing to potential sampling biases. For example, ^14^C dates of artefacts, charcoal and other materials indicate a single point in time of human occupation, but changing occupation intensity may or may not be reflected by the clustering of ^14^C dates of these artefacts (i.e. potential sampling bias). Furthermore, ^14^C dates of Thule or Dorset materials are unlikely to indicate the precise times of arrival and abandonment of the sites, because the very earliest and latest occupations are unlikely represented in samples submitted for dating. Studies based on continuous temporal records, such as dated lake or pond sediments adjacent to archaeological sites, potentially provide robust, more continuous and temporally coherent records of past occupations. Although limitations in sediment core records (owing to factors such as scouring, sediment hiatuses, dating uncertainties and diagenesis) may also limit sediment core reconstructions, sediment studies have the potential to enhance archaeological investigations.

Thus, we aimed to characterize the chemical and biological composition of pond sediment cores to determine whether we can observe evidence of historical occupation at strategically selected High Arctic ponds. We paired ponds directly adjacent to Thule dwellings constructed with whale bones to reference ponds located in the same region but with no archaeological evidence of human dwellings, settlement or use. Thus, we achieved an occupied/unoccupied site comparison for two sites to account for any non-human related or broader Arctic ecosystem changes. We hypothesized that the history of Thule, Dorset and Pre-Dorset occupations may be directly tracked by unique chemical signatures, coupled with more traditional palaeolimnological proxies, archived in dated pond sediments.

Sediments often provide a continuous record of past human occupation and, as such, stable isotopes in sediments are useful in tracking changes in human occupation [8,12]. δ^15^N profiles in pond sediments track nitrogen sources from trophically enriched sources, e.g. marine mammals compared with algae and plants [9]. For example, δ^15^N values in pond sediments from Thule-occupied sites increased at the time of their arrival [13]. The presence of the Thule people and nutrients from hunted animals also increased primary production in ponds, as evidenced by, for example, a shift to diatom species indicative of higher production and moss proliferation, as well as an increase in sedimentary chlorophyll *a* (chl *a*) during the time of Thule occupation [13]. Stable isotopes are thus useful tools for tracking changes in occupation over time as the arrival and/or departure of animals and/or humans are inferred by changes in the chemical composition of lake sediments.

Sediment biomarkers can help researchers track historical human occupations for archaeological studies. Prominent among these biomarkers are sterols and stanols, some of which are directly traceable to human occupation, and may thus directly track human influence on pond sediments [11,14]. Zoosterols (animal-derived) and phytosterols (plant-derived) are relatively stable in cold and anaerobic environments [15,16], and consequently fluctuations in zoosterols and phytosterols in lake sediments may be linked to human occupation (from the humans themselves and/or the mammals they kept or hunted), particularly in an otherwise barren and oligotrophic setting like an Arctic coastal environment. For example, cholesterol, identified in all mammals (i.e. humans, whales and seals, as relevant to this study), increased in High Arctic lake sediments as a result of wastewater input [15,17]. Coprostanol, produced from cholesterol in the guts of higher organisms, is elevated in human faeces [18], and is often much higher in wastewater-influenced lake sediments compared with reference lake sediments receiving no wastewater [19–22]. At remote, High Arctic sites, zoosterols naturally entering ponds are expected to be extremely low unless humans and domesticated species are present in a pond catchment [17]. In contrast, sitosterol is a phytosterol found in terrestrial and aquatic plants as well as phytoplankton [17,23,24] and consequently, sitosterol in lake sediments may track changes in allochthonous and/or autochthonous primary production [15]. Likewise, some metals, particularly cadmium (Cd), copper (Cu), lead (Pb) and zinc (Zn), bioaccumulate in whales and other marine life, and may be subsequently recorded in lake sediments as a result of human activity [25–28]. For example, Brimble *et al*. [29] calculated biogenic enrichment factors as the metal concentration in seabird-derived tissues relative to background sediment concentrations to identify metals most likely to be enriched in sediments near a coastal Arctic seabird colony. Accordingly, a similar approach of comparing metal concentrations in whale tissues relative to sediment background concentrations may help us identify metals likely to be enriched in sediments near sites occupied by Thule whalers. Therefore, we hypothesized that sterols, stanols and certain metals should increase in human-affected sediments, either directly from the humans themselves or from the marine and terrestrial mammals they brought to their residential sites.

**Figure 1.**
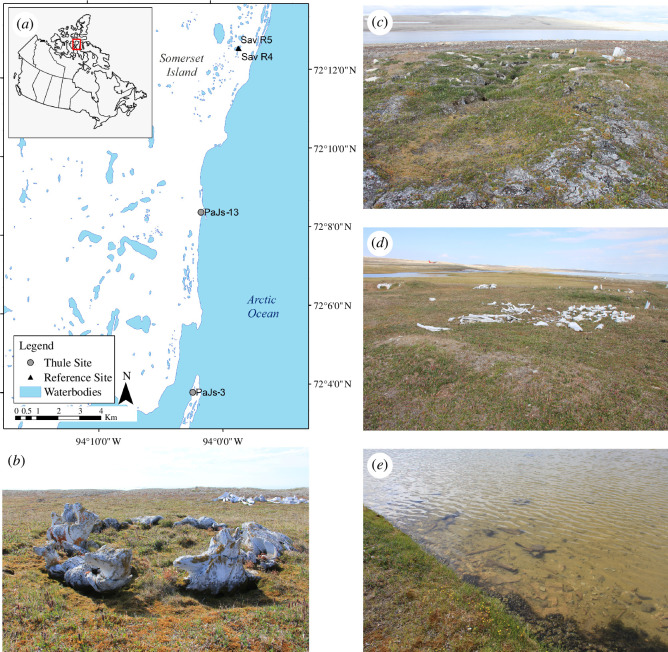
(*a*) Map of Thule-influenced ponds (PaJs-3 and PaJs-13) and reference ponds (Sav R4 and Sav R5) located on Somerset Island, Nunavut, Canada. The inset image is a map of Canada with Somerset Island outlined by a rectangle. (*b*) Circular arrangement of bowhead whalebones at PaJs-13. (*c*) Remnants of Thule structures at PaJs-3. (*d*) Arrangement of whalebones at PaJs-13. (*e*) Evidence of whalebones in the pond at PaJs-3. Photos: L.E.K.

Here, we reconstructed Dorset and Thule occupation history on Somerset Island in Canada’s High Arctic by examining the chemical and biological markers preserved in dated pond sediments (figure 1, electronic supplementary material, table S1). Specifically, we employed a multi-proxy approach, analysing pond sediments for sterols, stanols, stable nitrogen isotopes (δ^15^N), metals, chl *a* and diatoms. We strategically selected sites with clear evidence of major Thule occupation (see electronic supplementary material for details on the archaeological sites examined). PaJs-13 (electronic supplementary material, figure S1) consists of 23 semisubterranean whalebone dwellings and PaJs-3 (electronic supplementary material, figure S2) similarly consists of 22 semisubterranean whalebone dwellings. PaJs-13 had no evidence of Dorset occupation and at PaJs-3, a large Dorset site is located 6 km to the south of this site (see electronic supplementary material for more details). PaJs-3 is further divisible into a northern and southern site (see electronic supplementary material for details); this study focused on the northern site.

## 2. Material and methods

PaJs-13 and PaJs-3 (figure 1; electronic supplementary material, figures S1 and S2), located on Somerset Island in the Canadian High Arctic, are archaeological sites of significant Thule presence as evidenced by the high number of whalebone houses in the vicinity of the ponds [2]. Nine houses at each site consist of heavier bones, suggesting long-term occupation [2]. At PaJs-3, moss and whalebones were in the area immediately surrounding the pond, and the remainder of the landscape was barren rock. Conversely, vegetation and whalebones were present in a much larger radius surrounding PaJs-13.

Reference sites, Sav R4 and Sav R5 (figure 1), were selected based on their proximity to the archaeological sites, but with the absence of human-made structures and whalebones in the vicinity of the ponds. Detailed site information (latitudes, longitudes, surface area, height above sea level, nutrient concentrations in water) are in electronic supplementary material, table S1.

Surface water samples were collected near the shore of each pond in pre-rinsed (10% HNO_3_ for total metals and 10% H_2_SO_4_ for total phosphorus, soluble reactive phosphorus, particulate organic nitrogen, nitrate, dissolved organic carbon (OC) and dissolved inorganic carbon, among others) Nalgene bottles. Water samples for dissolved OC were filtered (Sartorius acetate filter, 47 mm and 0.45 µm) and stored at 4°C; water samples for particulate OC and particulate organic nitrogen were filtered onto pre-ashed filters (Whatman GF/F, 47 mm and 0.45 µm) and frozen until analysis. The National Laboratory for Environmental Testing, Burlington, Ontario, Canada, conducted all water chemistry analyses.

Lake sediment cores were dated by ^210^Pb, ^137^Cs and ^14^C. We collected sediment cores from the centre of each pond by pushing a 3-inch (7.6 cm) diameter tube into the sediments, which we subsequently sectioned into 0.5 cm intervals using a Glew extruder [30] and froze at −20°C. ^210^Pb dates were determined using an Ortec High Purity Germanium Gamma Spectrometer (Oak Ridge, TN, USA) at the University of Ottawa. Efficiency corrections were made using certified reference materials from the International Atomic Energy Association (Vienna, Austria). We constructed ^210^Pb and ^137^Cs profiles using the constant rate of supply (CRS) model [31] (ScienTissiMe; Barry’s Bay, Ontario, Canada). The CRS-derived ^210^Pb dates and the ^14^C ages were then interpolated together using the statistical package rbacon [32] to establish age-depth models for all cores (electronic supplementary material, figures S3–S8 and table S2).

To determine the radiocarbon dates, 1–3 mg OC of freeze-dried material was weighed into culture tubes. Anthracite and To-12586 were used as standards for background and 2910 ± 50 BP, respectively. We submerged the samples in 5 ml of 1 N HCl for 30 min in a metal bead bath set to 80°C, removed the HCl, added 5 ml milli-Q water, centrifuged the samples for 3 min at 2700 rpm, removed the water and freeze-dried the material. Samples were analysed on a 3 MV tandem accelerator mass spectrometer (High Voltage Engineering) at the AEL AMS Laboratory, University of Ottawa. Dates were calibrated using OxCal v. 4.3 [33] and the IntCal13 calibration curve [34]; we reported dates according to the conventions outlined by Millard [35]. Error estimates for ^14^C-derived dates were calculated using the ‘rbacon’ statistical package [32].

Bone processing for ^14^C and stable isotope determinations for ultrafiltered samples followed [36] and references therein. Briefly, aliquots of approximately 200 mg of cortical bone from the submitted samples (plus ^14^C-free blanks and known-age secondary standards) were crushed to mm-sized chips and decalcified overnight with 1 N HCl at room temperature. A measured amount of acid was used, calculated as just sufficient to dissolve all of the bone minerals if no collagen was present. The samples were then washed with ultrapure milli-Q water, gelatinized at pH 2 at 60°C overnight, and ultrafiltered with precleaned Vivaspin 15 Turbo ultrafiltration devices to select the >30 kDa molecular weight fraction, which was frozen and lyophilized overnight in a vacuum centrifuge. Aliquots of 2 mg of the lyophilized collagen for ^14^C dating were transferred to 6 mm quartz combustion tubes. CuO oxidizer and silver wire getter were added and the tubes were sealed under vacuum and combusted at 900°C. CO_2_ graphitization and ^14^C measurement by AMS were carried out at the Keck AMS laboratory at the University of California Irvine.

We analysed the pond sediments for sterols and stanols in accordance with the methodology outlined by Gallant *et al*. [17]. Briefly, 0.1 g of freeze-dried sediments was extracted in dichloromethane (high-grade, Optima brand) and separated using a 1 g LC-Si SPE column (Sigma-Aldrich, Oakville, ON, Canada). 100 μl of the sample was evaporated to dry, reconstituted in 100 μl of 99% N,O-bis(trimethylsilyl)trifluoroacetamide)+1% trimethylchlorosilane, and heated for 2 h at 60°C. An amount of 900 μl of toluene (high-grade, Optima brand) and 10 μl of 10 000 ng ml^−1^ of p-terphenyl-*d*_14_ (Cambridge Isotope Laboratories, Tewksbury, MA, USA) were added to the sample. Samples were analysed using an Agilent 6890 gas chromatograph–5973 mass selective detector in electron impact, selected ion monitoring (SIM) mode (Agilent 19091 J-433 HP-5 5% phenyl methyl siloxane 29.8 m × 250 μm × 0.25 μm column). Sterol concentrations were volume corrected to p-terphenyl-d_14_ using MSD ChemStation D.02.00.275. Additional dilutions were analysed, as required. The limit of quantification was set to a signal-to-noise ratio of three. All data analyses were conducted with R statistical computing environment (v. 3.5.2). Samples were recovery corrected to d6 cholesterol. Sterol and stanol concentrations below the limit of detection were corrected to MDL/√2 as determined by Gallant *et al*. [17]. Summary sterol and stanol concentrations are presented in electronic supplementary material, table S3.

We also collected periphyton and zooplankton samples in ponds and analysed them for sterols/stanols to determine whether other autochthonous sources may contribute sterols/stanols to sedimentary deposits. We sieved (125 µm) and filtered periphyton samples through a 110 mm Whatman glass microfiber filter (GF/F) (42.5 mm and 0.7 µm; pre-heated for 3 h at 400°C). The periphyton and filters were air-dried and homogenized before further analysis. Zooplankton samples were collected with a zooplankton net (63 μm mesh), concentrated and stored at 4°C. Sterols analysis in periphyton and zooplankton then followed the same protocols as sediment samples (above).

We analysed unacidified pond sediments for δ^15^N (‰ air), following the methodology outlined in Gallant *et al*. [37]. Analyses were conducted using a Micro Cube elemental analyser (analytical precision: ±0.2‰ using glutamic acid) at the Ján Veizer Stable Isotope Laboratory (formerly G. G. Hatch SIL Laboratory), located at the University of Ottawa, Ontario, Canada. We determined % OC on decalcified samples using the same instrument.

We analysed select pond sediments for 29 metal(loid)s using 0.5 g dry weight (dw) of sediment. The analysis was conducted using inductively coupled plasma mass spectrometry (prepared using an aqua regia digestion) at SGS Minerals Services, Lakefield ON, Canada; a lab accredited by the Canadian Association for Laboratory Accreditation. Metal concentrations below the MDL were replaced with MDL/√2 and then selected metals were normalized to the concentration of titanium to account for natural weathering [38]. Metals were normalized to titanium to minimize the effect of geogenic factors on concentrations. We calculated biogenic enrichment factors, a metric used in this instance to rank elements based on their likelihood of being enriched in sediment from the deconstruction of whales. These enrichment factors are calculated as the mean concentration of an element in whale tissue divided by the same element in background sediment from the region (see electronic supplementary material, table S4 for further details).

Chl *a* was analysed following methods in Michelutti *et al*. [39] and Michelutti & Smol [40]. Importantly, this method tracks trends in chl *a*, as well as its isomers and its main diagenetic products. Briefly, we freeze-dried enough sediments to cover the bottom of a 19 × 65 mm glass shell vial (Fisher cat. no. 03-339-26D); sediments were then sieved through a 125 μm mesh. Samples were analysed for spectral data between 400 and 2500 nm using a Model 6500 series Rapid Content Analyzer (FOSS NIRSystems.). Each sample represents an average of 32 scans. A ceramic reference standard was analysed between each sample. Sedimentary chl *a* concentrations were inferred using log-transformed data from Michelutti *et al*. [39] with the equation: chl *a* + derivatives = EXP (0.83784 × LN (peak area 650−700 nm) + (−2.48861)).

### (a) Statistical analyses

Palaeolimnological data were fitted using generalized additive models (GAMs) and procedures recommended by Simpson [41] to account for heteroscedasticity typical of these kinds of data. We identified periods of statistically significant change in our sediment proxies by identifying periods, where the first derivative of the GAM did not bound 0 [41]. These analyses were completed using the R packages mgcv v. 1.8-42 [42,43].

## 3. Results

### (a) Radiocarbon dating

A detailed description of our sediment dating results (electronic supplementary material figures S3–S8 and table S2) is given in electronic supplementary material. We gave special attention to two critical dates: 1200 CE, the expected date of Thule arrival and expansion, and 1500 CE, the expected approximate date of Thule site abandonment [2]. Our models dated 1200 CE to be represented in PaJs-3 at 19 cm (95% confidence interval (CI) from 1075 to 1257 CE based on rbacon model uncertainties) and in PaJs-13, 1200 CE was at 14 cm (95% CI: 1061–1314 CE). CIs for this critical date spanned between 80 and 250 years in our Thule-affected ponds. Error estimates for 1200 CE were larger in our reference ponds that had much lower sediment accumulation rates. For example, Sav-R4 had no intervals representing 1200–1500 CE because all dated intervals, including their CIs, did not overlap this range (electronic supplementary material, figure S7). SAV-R5 had better dating resolution, where 1200 CE was observed at 8.5 cm, though with large dating uncertainty (95% CI: 873–1580 CE). Likewise, our models dated 1500 CE, to be represented in PaJs-3 at 16.5 cm (95% CI: 1302–1681 CE) and in PaJs-13 at 10 cm (95% CI: 1393–1585). We dated 1500 CE in SAV-R5 at 7 cm (95% CI: 1159–1766 CE).

### (b) PaJs-3

**Figure 2.**
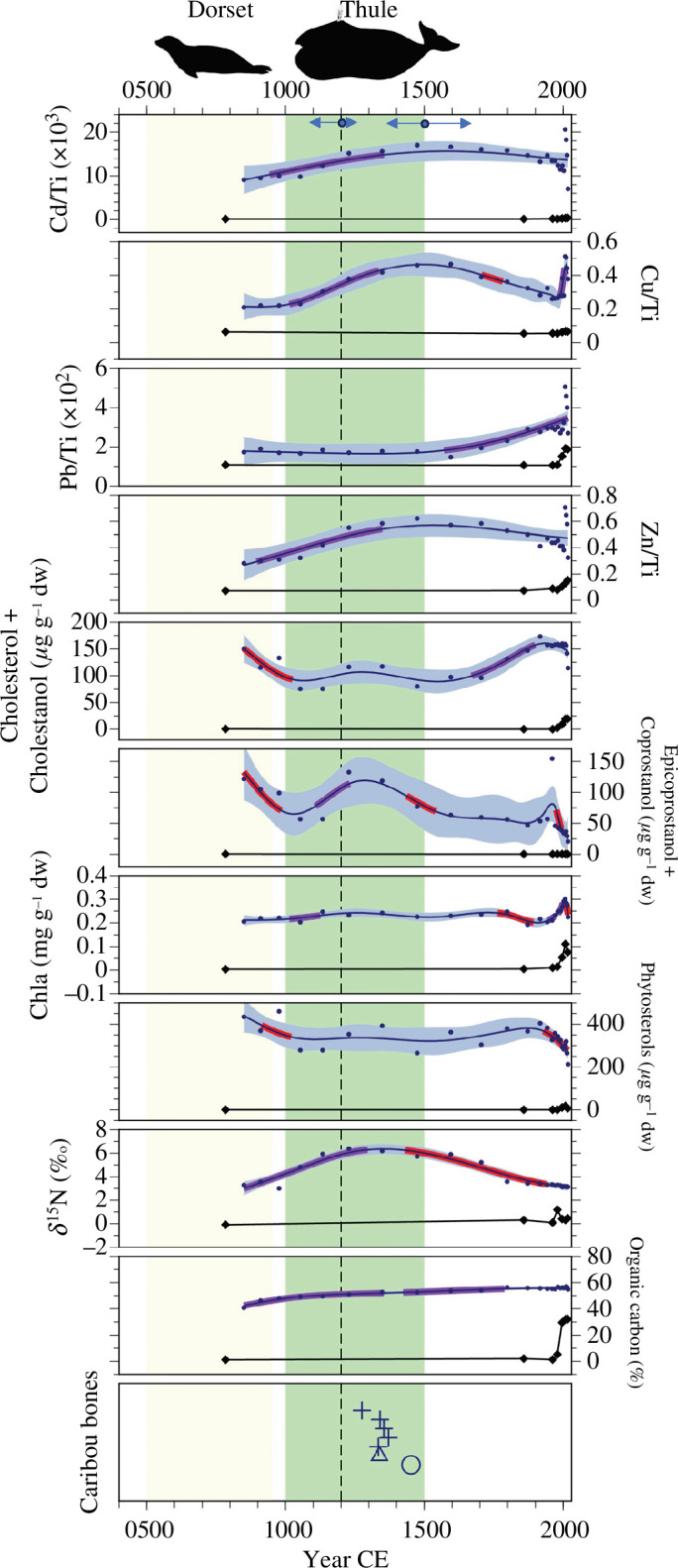
Metals (normalized to titanium), sterols and stanols (μg g^−1^ dw), chl *a* (mg g^−1^ dw), δ^15^N (‰) and percentage OC profiles in a Thule-influenced pond (PaJs-3; circles) and a reference pond (Sav R4; diamonds). The green and yellow shaded areas denote our suggested time of Thule and Dorset occupation, respectively. Significant increases based on GAMs are shown in purple, whereas significant decreases are shown in red. The dashed vertical line represents the previously suggested time of Thule occupation (1200 CE). The two-directional arrows in the top panel mark the 95% CIs in ^14^C dates for 1200 CE and 1500 CE based on rbacon estimates (see text).. Phytosterols include the sum of sitosterol, stigmastanol and campesterol. The caribou bones panel illustrates the age of ^14^C-dated caribou bones, where each shape denotes a different feature (i.e. whalebone house) and each point denotes a different caribou bone. For example, the five ‘+’ symbols indicate that there were five different caribou bones dated from the same site.

This pond is currently elevated in nutrients (total phosphorus = 29 μg l^−1^), as are other ponds in this study (electronic supplementary material, table S1). However, evidence of higher past nutrient concentrations is evident. Concentrations of cholesterol + cholestanol and the human dominant coprostanol + epicoprostanol increased after *ca* 1000 CE (95% CI: 929–1107 CE) corresponding approximately to Thule arrival, although these sterols also reached elevated concentrations earlier during the Dorset period (figure 2; electronic supplementary material, figure S9). These sterol concentrations greatly exceeded those in sediments from reference sites Sav R4 and Sav R5 (electronic supplementary material, table S3 and detailed below) providing strong evidence of human occupation during both the Dorset and Thule periods.

A total of 29 metals were analysed; however, we focused our analysis on cadmium (Cd), copper (Cu), lead (Pb) and zinc (Zn) (electronic supplementary material, tables S4–S6), as these metals are commonly recorded in lake sediments as a result of human activity [25–27], and are bioaccumulated in whales [44,45]. Cd and Cu increased coeval with δ^15^N values (figure 2) and zoosterols during Thule occupation (ca 1000–1500 CE), though these metals also peaked in twentieth-century deposits during widespread atmospheric contamination. Lead, however, remained stable throughout most of the sediment core, but then also peaked in twentieth-century sediments.

We observed significant decreases (based on GAMs) in cholestanol + cholesterol, epicoprostanol + coprostanol and phytosterols from *ca* 850 (95% CI: 755–927)−1000 (929–1107) CE. The most pronounced increases in sediment proxies were between *ca* 1000 and approximately 1300 (1226–1495) CE, which were most evident for Cd/Ti, Cu/Ti, Zn/Ti, epicoprostanol + coprostanol and δ^15^N based on GAM results, marking the most likely period for the rise in Thule occupation (figure 2). Declines in epicoprostanol + coprostanol and δ^15^N at *ca* 1500 (1328–1751)−1700 (1573–1855) CE were evident following the Thule occupation, as were later declines in Cu/Ti and chl *a* from *ca* 1700 to 1900 (95% CI for 1900: 1841–1944) CE (figure 2).

Percentage OC (%OC) was generally elevated in PaJs-3 (electronic supplementary material, table S7), ranging from approximately 41% to 57%. Percentage OC increased before *ca* 1000 CE and remained elevated into present-day deposited sediments. Likewise, δ^15^N values increased nearly coeval with %OC (*ca* 1100 (988–1189) CE) and decreased *ca* 1800 (1720–1898) CE (figure 2).

Seven caribou bones found distributed at PaJs-3 were radiocarbon dated and ranged between 1275 and 1452 CE (mean 1352 ± 53 SD, figure 2, electronic supplementary material, table S9); thus, all caribou bones coincided with the Thule occupation of this site (figure 2).

### (c) PaJs-13

**Figure 3.**
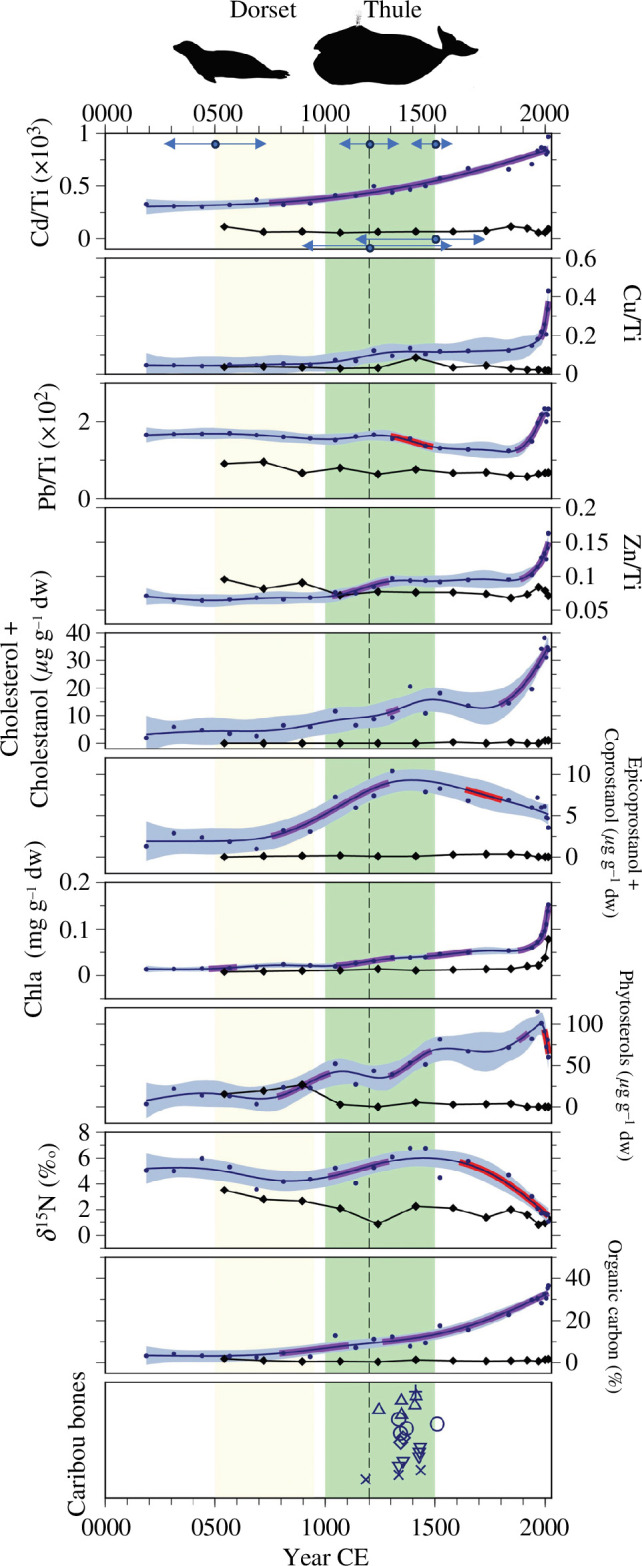
Metals (normalized to titanium), sterols and stanols (μg g^−1^ dw), chl *a* (mg g^−1^ dw), δ^15^N (‰) and %OC in a Thule-influenced pond (PaJs-13; circles) and the reference pond (Sav R5; diamonds). The green and yellow shaded areas denote our suggested time of Thule and Dorset occupation, respectively. Significant increases based on GAMs are shown in purple, whereas significant decreases are shown in red. The dashed vertical line represents the previously suggested time of Thule occupation (1200 CE). The two-directional arrows in the top panel mark the 95% CIs in ^14^C dates for 1200 CE and 1500 CE based on rbacon estimates (upper arrows are for PaJs-13 and the lower arrows are for Sav R5; see text). Phytosterols include the sum of sitosterol, stigmastanol and campesterol. The caribou bones panel illustrates the age of ^14^C-dated caribou bones, where each shape denotes a different feature (i.e. caribou bones from within a whalebone house) and each point denotes a different caribou bone. For example, the three ‘x’ symbols indicate that there were three different caribou bones dated from the same site.

In PaJs-13, total phosphorus was 12 μg l^−1^, showing lower enrichment than that in PaJs-3 (electronic supplementary material, table S1). The sediment proxies Cd/Ti, epicoprostanol + coprostanol, phytosterols and organic C (%OC) began to increase significantly in sediments pre-dating Thule occupation (*ca* 800 CE), and continued to increase well into the period of Thule occupation (figure 3). Other indicators of human occupation at this site (Cd/Ti, Zn/Ti and δ^15^N) began increasing significantly after 1000 CE, and others (cholestanol + cholesterol, phytosterols) increased *ca* 1200 CE (figure 3; electronic supplementary material, figure S11), which approximates other estimates [2] for Thule occupation in these locations (typically *ca* 1200 CE). Coprostanol + epicoprostanol peaked between *ca* 1000 and *ca* 1500 CE, and cholestanol + campesterol continued to increase into present-day sediments (figure 3; electronic supplementary material, figure S11). Most sterols increased during the Thule occupation; however, cholesterol did not increase until after *ca* 1800 (95% CI: 1768–1913) CE, and the phytosterols (sitosterol and stigmastanol) were not detectable throughout the sediment core (figure 3; electronic supplementary material, figure S11). Coprostanol + epicoprostanol was elevated during the Dorset and Thule occupation, then declined sharply in sediments deposited post-1500 (1393–1585) CE (figure 3), although not to pre-Thule values.

We also examined sterols in periphyton and zooplankton from the ponds to determine how they may have contributed to sterols in sediments. Zooplankton were most elevated in cholesterol (approx. 10 000 μg g^−1^ dw), making them a potential source of cholesterol to sediments (electronic supplementary material, figure S12); however, neither zooplankton nor periphyton contained appreciable coprostanol or epicoprostanol, confirming that these sterols likely originated from other sources. Zooplankton were also elevated in the phytosterols sitosterol and stigmastanol (electronic supplementary material, figure S12).

Metals (Cd, Cu, Pb and Zn, all relative to Ti) in PaJs-13 sediments were generally low prior to *ca* 1000 CE but increased coeval with the Thule occupation, although their increase was more muted than in PaJs-3. Their highest values were reached in twentieth-century sediment deposits coinciding with atmospheric contamination of these metals, peaking at 9.7 × 10^–4^, 0.43, 0.02 and 0.16, respectively.

In PaJs-13, %OC increased slightly *ca* 1000 (884–1082) CE, just before the archaeological estimates for the arrival of the Thule people, and then more notably *ca* 1800 (1768–1913 CE). δ^15^N measurements, however, were elevated during both Dorset and Thule occupation, and decreased *ca* 1800 CE (figure 3). The large fluctuations in %OC affected sterol concentrations when normalized to sediment OC in both human-affected lakes (electronic supplementary material S13), but we noted that coprostanol and epicoprostanol were both elevated during the Dorset and Thule periods relative to the reference lakes and the period following occupation, with the exception of one anomalous interval (electronic supplementary material S13). Sterol and stanol ratios in the human-influenced ponds were generally uninformative in part because some of the constituents were undetectable (electronic supplementary material, figure S14).

Chl *a* concentrations first rose between *ca* 500 (276–760) CE and *ca* 1000 (884–1082) CE, coincident with the arrival and departure of the Dorset people (figure 3). This was followed by a more pronounced rise *ca* 1000 CE, which slightly precedes the estimated arrival of the Thule. Chl *a* ultimately peaked in sediments deposited in the late twenty-first century.

The twenty ^14^C dated caribou bones collected from PaJs-13 spanned between 1185 CE and 1510 CE (1368 ± 71 CE, figure 3, electronic supplementary material, table S9).

### (d) Sav R4

Concentrations of sterols and stanols in Sav R4 (figure 2; electronic supplementary material, figure S9) were lower than the influenced pond sediments. In most instances, the concentration of sterols and stanols in the reference ponds did not exceed the method detection limit (MDL), specifically, only campesterol, cholesterol, coprostanol and epicoprostanol exceeded the MDLs. Sterol accumulation rates were also much lower in SavR4 than in both of the Thule-affected pond sediments (electronic supplementary material, table S3). Like sterols and stanols, metals (relative to Ti) were generally stable throughout the sediment cores, with only slight increases in Pb and Zn in the most recently deposited sediments. Likewise, %OC (0.5–32%), was very low throughout the sediment core, until it peaked in the surface sediments (figure 2). δ^15^N values in Sav R4 was stable throughout the sediment core, ranging from −5.3% to 1.2‰ (electronic supplementary material, figure S9). Summary stable isotope data are presented in electronic supplementary material, table S7. In Sav R4, chl *a* was relatively low and constant through the sediment core until the late 1900s, when chl *a* increased. Chl *a* concentrations in Sav R4 sediments were always lower than those recorded in the influenced sediments from PaJs-3 and PaJs-13.

### (e) Sav R5

Sterol and stanol concentrations in Sav R5 sediments were lower than the concentrations recorded in human-influenced pond sediments (figure 3; electronic supplementary material, figure S11). Specifically, only coprostanol and epicoprostanol exceeded the MDLs. Cholestanol was not detected limiting the use of some ratios for tracking humans. Likewise, metal ratios and %OC (ranging from 0.3% to 2.2%) were low and stable throughout the sediment cores. In fact, Sav R5 was the only pond in which OC did not increase (or in the case of PaJs-3, was not already elevated) in recently deposited sediments. δ^15^N measurements ranged from 0.8% to 3.5‰ in Sav R5 sediments, with slightly higher values during Dorset occupation relative to recently deposited sediments. Chl *a* concentrations were low and constant until the late 1900s, at which point chl *a* increased, peaking at 0.08 mg g^−1^ dw. Chl *a* concentrations in Sav R5 sediments were always lower than those recorded in PaJs-13 and PaJs-3 sediments.

## 4. Discussion

Sterols and stanols were consistently higher in human-influenced ponds relative to reference ponds. For example, the minimum concentration of cholesterol + cholestanol in PaJs-3 always exceeded the maximum concentration in the reference ponds (figures 2 and 3). Campesterol was only detected in one sediment layer of one of the reference ponds (Sav R4), while campesterol was detected throughout the majority of both sediment cores adjacent to the archaeological sites with concentrations often exceeding 1 μg g^−1^ dw (electronic supplementary material, figure S9). Similarly, cholestanol was not detected in either reference pond, but was present in nearly all sediment intervals of the influenced ponds. Cholesterol, coprostanol and epicoprostanol were low in reference ponds, and detected only in twentieth-century deposits. We suspect that these low concentrations in surface sediment reflect the small surface organic layer, which is likely composed of decaying zooplankton and phytoplankton. Indeed, cholesterol concentrations were also very low in phytoplankton and zooplankton of Sav R4 (electronic supplementary material, figure S12). The small surface sediment concentrations of coprostanol and epicoprostanol in Sav R4 may also be the result of *in situ* degradation of cholesterol [44]. In PaJs-3, increased phytosterol concentrations (figure 2) in sediments deposited post-Thule occupation suggested sustained productivity following Thule departure, as further supported by elevated chl *a* concentrations (figure 2). Sustained zoosterol and phytosterol concentrations in post-Thule deposited sediments may also have resulted from continued shoreline run-off from carcasses, faecal matter or moss substrates, as evidenced by sustained epicoprostanol + coprostanol concentrations (figures 2 and 3), thus, supplying a continued source of sterols and stanols to the sediments. Given the elevated concentrations of sterols and stanols in PaJs-3 and PaJs-13 during Thule and Dorset occupation periods, and the general absence of sterols and stanols in the reference ponds, we infer that sterols and stanols, in both proportion and presence, recorded the presence of the Thule and Dorset people in these high Arctic pond sediments.

Apart from small quantities of zoosterols originating in zooplankton within ponds (electronic supplementary material, figure S12), natural sources of zoosterols to Arctic watersheds are probably minuscule relative to human inputs and those from domesticated animals. Terrestrial Arctic animals that may be present within these pond watersheds are Arctic fox, lemming, Arctic hare and polar bear. A recent study that looked at distributions of 5β-stanols in Arctic and domesticated animal faecal material found that coprostanol was dominant in omnivore/carnivore faeces at >50% composition in dogs and humans, and ~10% in herbivores [46]. Furthermore, coprostanol was by far the dominant sterol driving the ‘fingerprint distinction’ between omnivores and herbivores, followed by epicoprostanol. Thus, although herbivores may be a small source of coprostanol to Arctic sediments, coprostanol can be confidently used to identify humans and dogs along these shorelines. Other potential mammalian carnivores, like foxes and polar bears, are more solitary than humans and their dogs and would be far less likely to produce detectable amounts of these sedimentary sterols. The early increase in multiple proxies in the influenced ponds suggests that the Thule people may have arrived at PaJs-3 and PaJs-13 approximately 100–200 years before the previously suggested date of 1200 CE based on the dating of artefacts, even when accounting for dating uncertainties in these cores (electronic supplementary material, figures S5, S6). In both PaJs-3 and PaJs-13, sterols and stanols increased *ca* 1000 CE, coincident with increases in δ^15^N measurements, as well as Cd, Cu and Zn (figures 2 and 3; electronic supplementary material, figures S9 and S11). In these ponds, we attributed the elevated zoosterol concentrations beginning in *ca* 1000 CE to the flensing of bowhead whales in these near-shore sites, along with the construction of homes and the release of human waste within the pond catchments. In addition, Thule are known to have kept dogs [47], which also produce zoosterols including higher quantities of coprostanol [18,46]. In PaJs-3, the increase in zoosterol concentrations occurred coeval with an increase in phytosterol concentrations. We hypothesized that the bowhead whales and other hunted animals contributed additional nutrients to the pond and surrounding landscape, which stimulated primary productivity. This hypothesis is supported by the rise in sediment chl *a* values, which fluctuated in concordance with changes in the concentrations of phytosterols, thus tracking primary production during the period of Thule occupation (figure 2).

While a more recent date of 1200 CE has been proposed for the arrival of the Thule people [2,45,48], this later date is based on ^14^C dated whalebones and caribou and thus may not completely encompass the time of Thule occupation because the dates are dependent on the bones selected for dating. It is unlikely that the bones of the first and last animals hunted were located by archaeologists and made available for dating. Thus, while our ^14^C dated caribou bones from both PaJs-3 and PaJs-13 all date after 1200 CE (with the exception of one bone from PaJs-13: 1185 CE), it is possible that the earliest caribou hunted at the site were not included in the sample, or perhaps caribou hunting may not have been active in the earliest days of Thule occupation. Conversely, given errors associated with ^14^C dating, the long ^14^C reservoir age at these locations, and the low sedimentation rates in these ponds, precise dating (± <100-year error) is unlikely. Sediment cores do, however, provide the advantage of a more robust and continuous record than bone fragments or records based on other artefacts, and unlike archaeological artefacts, may extend prior to the historical period of interest.

Although structures belonging to the Thule people were present at both PaJs-13 and PaJs-3, we observed marked differences in the presence, absence and abundance of sterols and stanols between the two sites. We suggest that site size and distribution of artefacts affected the chemical profiles of sediment cores. First, sterol and stanol concentrations in PaJs-3 sediments were generally 10-fold higher than those in PaJs-13 sediments. Sitosterol and stigmastanol were not detected within sediments from PaJs-13. The absence of these phytosterols was unexpected because the area immediately surrounding PaJs-13 was rich in vegetation and %OC was elevated (maximum of 36%), suggesting elevated primary production [49]. Elevated sitosterol and stigmastanol is anticipated in productive ponds owing to the increase in abundance of algae [50] and herbivore faeces [23], respectively. However, %OC was also elevated in the reference pond, Sav R4 (maximum of 32%), suggesting that increased %OC alone may not be associated with elevated sterol concentrations. (Sav R4 also had TP = 28 μg l^−1^, similar to Thule-affected ponds, electronic supplementary material, table S1). Thus, we attributed the absence of a strong sterol signal in sediments from PaJs-13 to site size; PaJs-13 is a large site with lower TP (12 μg l^−1^; electronic supplementary material, table S1), with whalebones spread for tens of meters beyond the perimeter of the pond itself. By comparison, PaJs-3 (TP = 29 μg l^−1^; electronic supplementary material, table S1) is a relatively isolated pond where whalebones were concentrated in the immediate area, and the perimeter of vegetation surrounding the pond was much smaller than that observed at PaJs-13. Second, only cholestanol and coprostanol were recorded in Dorset-influenced sediments from PaJs-13, whereas all analysed sterols and stanols were present in Dorset-influenced sediments from PaJs-3 (figures 2 and 3; electronic supplementary material, figures S9 and S11). We attributed the lower and/or absent sterol and stanol concentrations in PaJs-13 sediments to its larger size (see electronic supplementary material S1 and S2). We suspect that the Thule signal, and most notably the Dorset signal were diluted over the larger pond and site area of PaJs-13, whereas the sterols and stanols at PaJs-3 were concentrated around the smaller pond. Furthermore, the Dorset people typically hunted smaller mammals, such as fox and seal, and their remains were absent in the ponds. Conversely, the Thule mainly hunted bowhead whales, whose bones were still apparent in these ponds at the time of sampling. Therefore, we suggest that the Dorset people at these ponds had a smaller relative impact on these ponds as compared with the Thule, and that this already small signal of Dorset occupation was diluted within the larger site of PaJs-13.

We used multiple proxies in lake sediments to estimate the departure of the Dorset people from the near-shore ponds. In PaJs-3, cholesterol, coprostanol and epicoprostanol were elevated in sediments deposited *ca* 860 CE, and thus prior to Thule occupation (figure 2), suggesting influence from occupation by the Dorset who pre-dated the Thule at this location [4]. Relative sea level curves at this location showed that PaJs-3 was above sea level at the time of Dorset occupation, and the presence of Dorset winter houses and longhouses confirmed their presence within 6 km of the site [4]. Therefore, elevated sterol and stanol concentrations at *ca* 860 CE may reflect the historical occupation by the Late Dorset people (450−950 CE) [4]. The subsequent decrease of sterols and stanols in sediments at *ca* 995 CE and the subsequent increase in the post-*ca* 1000 CE sediments marks the approximate timing of the transition from Dorset to Thule occupation [4]. Because the sediment core from PaJs-3 does not pre-date Dorset occupation, we are unable to observe a change in δ^15^N and %OC values with the arrival of the Dorset (before *ca* 450 CE). Instead, δ^15^N and %OC values were similar in sediments deposited during Thule and Dorset occupation, which further supports a human influence during these two time periods. In PaJs-13, concentrations of cholestanol and coprostanol increased toward the bottom of the sediment core (*ca* 100 BCE), which closely matched the earliest recorded arrival of the Dorset people to the High Arctic in *ca* 450 BCE [4]. δ^15^N values increased coeval with cholestanol and the sterol ratios, and a small increase in chl *a* was also recorded during the period of Dorset occupation. These independent proxies lend further evidence that PaJs-13 was occupied by the Dorset people prior to the Thule people.

Human practices have altered the metal composition in the environment for thousands of years. We thus examined the concentrations of Cd, Cu, Pb and Zn normalized to Ti in the four ponds (figures 2 and 3) to determine if metal concentrations tracked human occupation. Cd, Cu and Zn were three of the most concentrated metals in bowhead whale livers relative to their geological background values [28] (electronic supplementary material, table S4); thus, we expected the flensing of bowhead whales in High Arctic ponds to increase these sediment metal concentrations. This pattern was indeed confirmed by our results; Cd, Cu and Zn increased in sediments between *ca* 1000 and 1500 CE in PaJs-3 and PaJs-13, coinciding with the occupation of the Thule people (figures 2 and 3; electronic supplementary material, table S4). Whereas we observed an increase in sediment metals during Thule occupation at both PaJs-3 and PaJs-13, sediment metals did not increase during Dorset occupation. This difference may be attributed to the distinct hunting practices of the two cultures [6]. The absence of small mammal bones in the ponds and surrounding area also suggests that the Dorset had a smaller effect on these ponds. We suggest that the higher metal concentrations (relative to Ti) found in PaJs-3 relative to PaJs-13 could again be attributed to community size relative to the size of the ponds. We attributed the higher metals in PaJs-3 sediments to a more focused input from the smaller site and the lower metals in PaJs-13 to dilution from a much larger site. Cadmium and zinc were the two metals most enriched in whale tissues relative to sediment background (electronic supplementary material, table S4), and they were the same metals to show highest enrichment in sediment during Thule occupation in both inhabited sites (electronic supplementary material, table S4). Surface sediment peaks in Cd, Cu, Pb and Zn were reflective of twentieth century increases in atmospheric emissions from mining and fossil fuel combustion [51–53].

## 5. Conclusion

Our study highlights the benefit of combining archaeological approaches with palaeolimnological methodologies to better refine historical periods of human occupation. We showed that multiple proxies in pond sediments can complement our understanding of Indigenous history and push the boundaries of traditional archaeological studies. This blending of fields and methodologies strengthened our interpretation of the timing of Thule and Dorset occupation in the High Arctic and demonstrated the capacity of these studies to further track long-term human interactions with the environment. Before this study, evidence of Dorset occupation at PaJs-13 and PaJs-3 was lacking. Through our multi-proxy analysis of pond sediments, we confirmed the presence of Dorset people at both these ponds. Specifically, we quantified the impact of human activity on pond sediment chemistry by comparing human-influenced to reference pond sediments. Our multiple independent proxies also suggest Thule arrival at these sites at *ca* 1000 CE, which pre-dates current estimates. The major indicators of human occupation were metals, sterols and stanols in Thule-influenced sediments relative to reference pond sediments. We also demonstrated that biotic enrichment factors were useful for tracking metal concentrations in human-influenced sediments (electronic supplementary material, table S4). Historical occupation of these High Arctic ponds was accompanied by higher chl *a* concentrations and diatom to cyst ratios, as expected with the fertilizing effects of human occupation. This study provided a first glimpse at changes in the sterol/stanol composition of lake sediments as a result of Thule and Dorset occupation in High Arctic ponds. The combined analysis of multiple independent proxies (including sterols, stanols, stable isotopes, metal ratios, chl *a*, diatoms and chrysophytes) in waterbody sediments may be useful to identify other historically important sites and track the timing of human presence in other locations.

## Data Availability

Data associated with this manuscript are available in the electronic supplementary material [54].
